# The formation of habits in the neocortex under the implicit supervision of the basal ganglia

**DOI:** 10.1186/1471-2202-16-S1-P212

**Published:** 2015-12-18

**Authors:** Meropi Topalidou, Daisuke Kase, Thomas Boraud, Nicolas P Rougier

**Affiliations:** 1 INRIA Bordeaux Sud-Ouest, Bordeaux, France; 2 Université de Bordeaux, CNRS UMR 5293, IMN, France; 3 LaBRI, Université de Bordeaux, IPB, CNRS, UMR 5800, Talence, France; 4 Laboratoire Franco-Israélien de Neurosciences, CNRS Bordeaux, Talence, Bordeaux, France

## 

**Figure 1:**
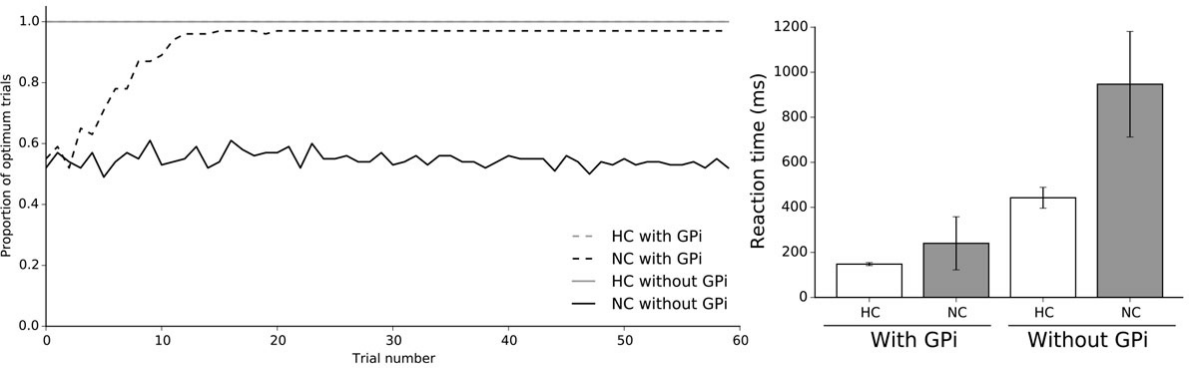
**Left. In habitual condition (HC), performances are optimal, with or without GPi. In novel condition (NC), only the intact model (with GPi) is able to learn the new stimuli while lesioned model performances stay at the level f chance**. **Right**. Analysis of the data shows that reaction time is higher in normal condition as compared to habitual condition with active and inactive GPi. The later increases significantly the reaction time in both conditions. All data have been averaged over 250 simulations.

If basal ganglia are widely accepted to participate in the high-level cognitive function of decision-making, their role is less clear regarding the formation of habits [1,2]. One of the biggest problem is to understand how goal-directed actions are transformed into habitual responses, or, said differently, how an animal can shift from an action-outcome (A-O) system to a stimulus-response (S-R) one, while maintaining a consistent behavior. We introduce a computational model (basal ganglia, thalamus and cortex) that can solve a simple two arm-bandit task using reinforcement learning and explicit valuation of the outcome [3]. Hebbian learning has been implemented at the cortical level such that the model learns each time a move is issued, rewarded or not. Then, by inhibiting the output nuclei of the model (GPi), we show how learning has been transferred from the basal ganglia to the cortex, simply as a consequence of the statistics of the different choices (see Figure 1). Because best (in the sense of most rewarded) actions are chosen more often, this directly impacts the amount of Hebbian learning and lead to the formation of habits within the cortex. These results have been confirmed in monkeys doing the exact same task where the BG has been inactivated using muscimol. This tends to show that the basal ganglia implicitly teach the cortex in order for it to learn the values of new options. In the end, the cortex is able to solve the task perfectly, even if it exhibits slower reaction times.
